# Supplementary material for: Two New Components from an Association of Marine Sponges Poecillastra sp. and Jaspis sp. and Their Inhibitory Effects on Biomarkers for Benign Prostatic Hyperplasia
Source: Mar Drugs. 2023 Sep 14;21(9):491. doi: 10.3390/md21090491 (PMC10532625; doi:10.3390/md21090491)
Supplement: Supplementary file 1 [file marinedrugs-21-00491-s001.zip › marinedrugs-2613068-supplementary.pdf]

Supplementary Materials

Two New Components from an Association of Marine Sponges *Poecillastra* sp. and *Jaspis* sp. and Their Inhibitory Effects on Biomarkers for Benign Prostatic Hyperplasia

Buyng Su Hwang [1], Sangbum Lee [2], Eun Ju Jeong [3,*] and Jung-Rae Rho [2,*]

[1] Bio-Resource Industrialization Center, Nakdonggang National Institute of Biological Resources, Sangju 37242, Republic of Korea

[2] Department of Oceanography, Kunsan National University, Kunsan 54150, Republic of Korea

[3] Department of Plant & Biomaterials Science, Gyeongsang National University, Jinju 52725, Republic of Korea

* Correspondence: jeong.ej@gnu.ac.kr (E.J.J.); jrrho@kunsan.ac.kr (J.-R.R.);
Tel.: +82-55-751-3224 (E.J.J.); +82-63-469-4606 (J.-R.R.)

Contents

Figure S1. HRESIMS for **1**. 2

Figure S2. The ^{1}H (A) and ^{13}C (B) NMR spectra for **1**. 3

Figure S3. COSY NMR spectrum for **1**. 4

Figure S4. TOCSY NMR spectrum for **1**. 5

Figure S5. HSQC NMR spectrum for **1** (blue: CH_2, black: CH_3 + CH). 6

Figure S6. HMBC NMR spectrum for **1** ($^{n}J_{CH}$ = 6 Hz). 7

Figure S7. NOESY NMR spectrum for **1**. 8

Figure S8. Expanded HETLOC NMR spectrum for **1**. 9

Figure S9. HRESIMS (A) and MSMS (B) data for **2**. 10

Figure S10. The ^{1}H (A) and ^{13}C (B) NMR spectra for **2**. 11

Figure S11. DQFCOSY NMR spectra for **2**. 12

Figure S12. TOCSY NMR spectra for **2**. 13

Figure S13. HSQC NMR spectrum for **2** (blue: CH_2, black: CH_3 + CH). 14

Figure S14. HSQC-TOCSY NMR spectrum for **2** (blue: $^{1}J_{CH}$, black: $^{n}J_{CH}$). 15

Figure S15. HMBC NMR spectrum for **2** ($^{n}J_{CH}$ = 8 Hz). 16

Figure S16. NOESY (up) and NOESY 1D (down) NMR spectrum for **2**. 17

Figure S17. Expanded HECADE NMR spectrum for **2**. 18

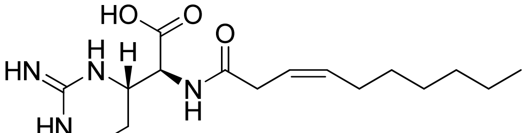

1

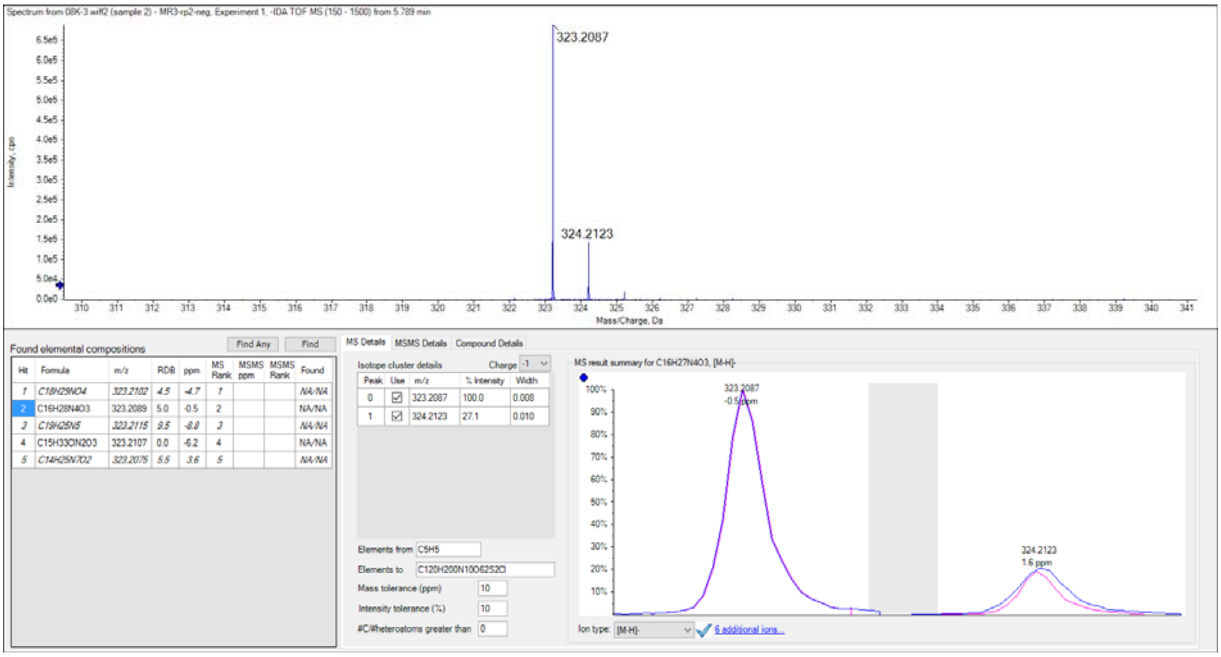

Figure S1. HR ESI MS data for **1**.

(A) 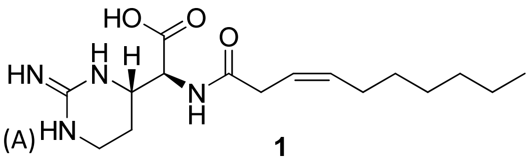

1

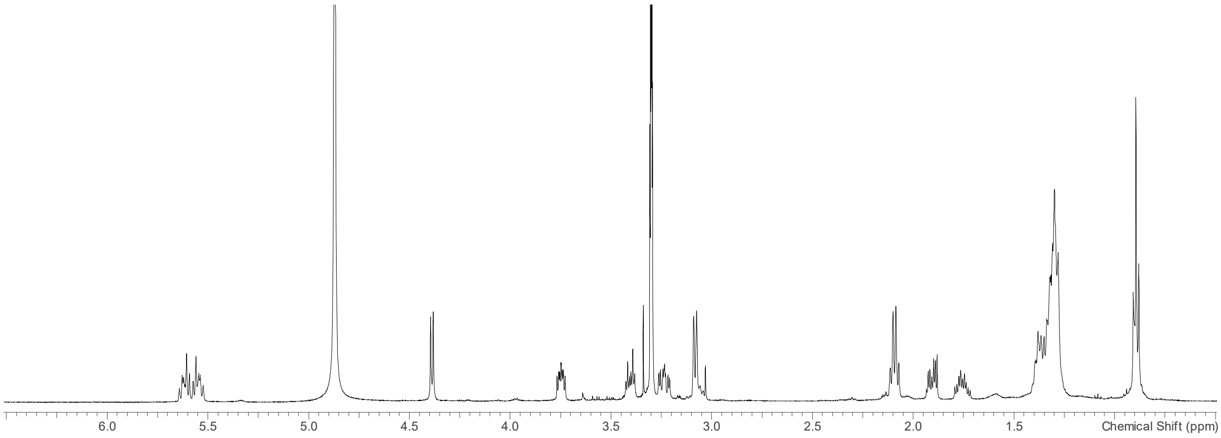

(B)

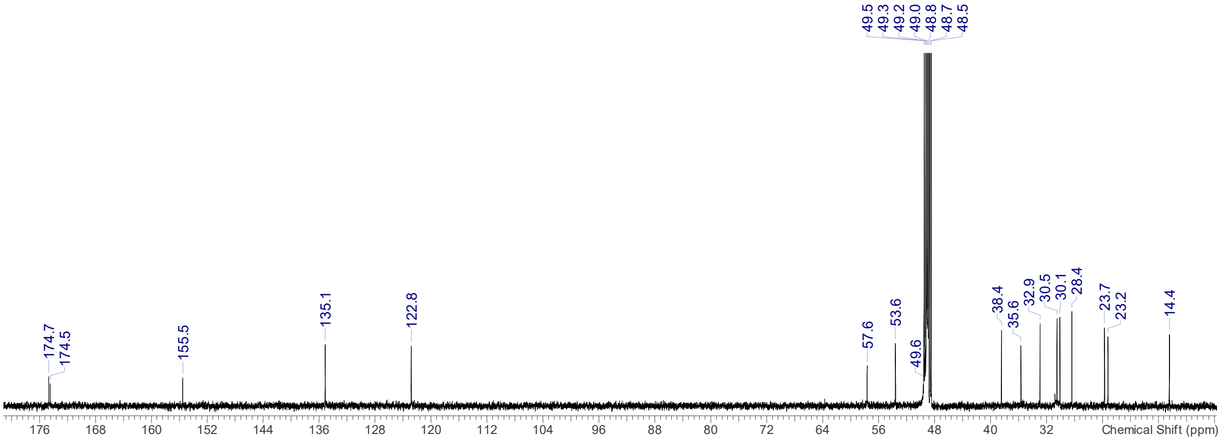

Figure S2. The ^{1}H (A) and ^{13}C (B) NMR spectra for **1**.

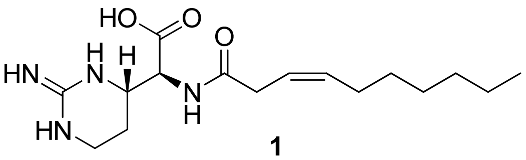

1

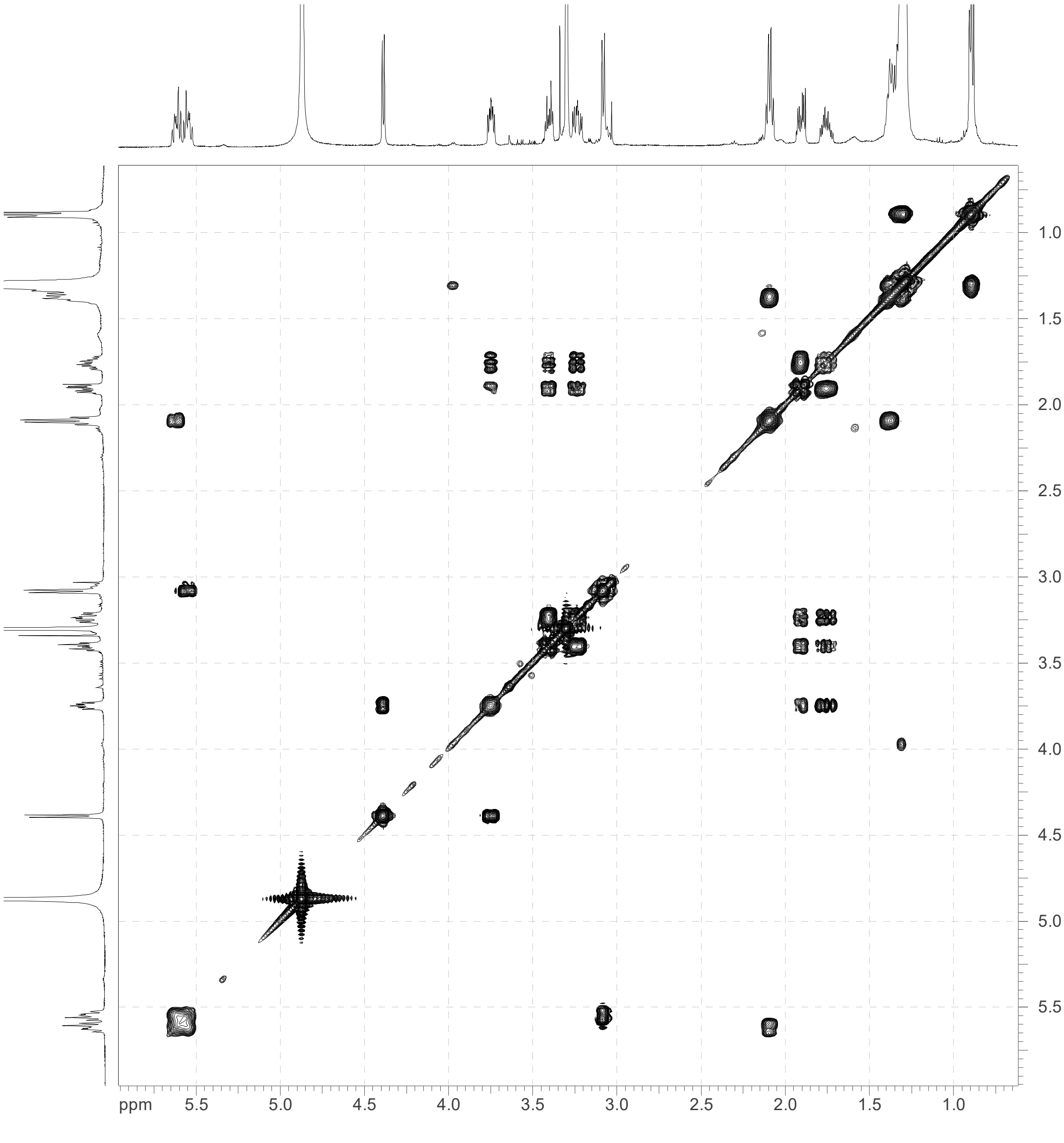

Figure S3. COSY NMR spectrum for **1**.

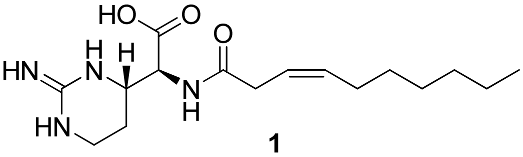

1

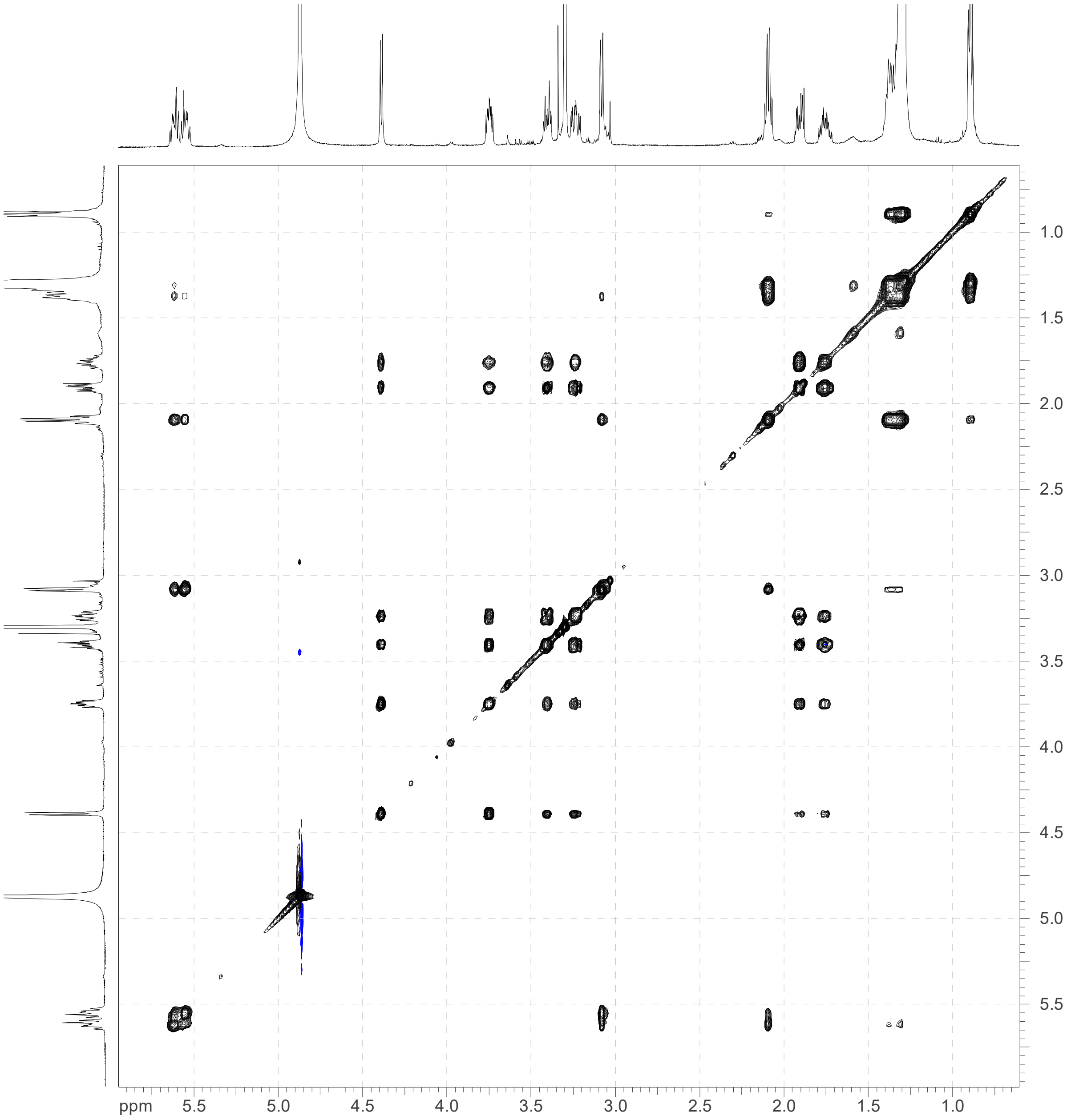

Figure S4. TOCSY NMR spectrum for **1**.

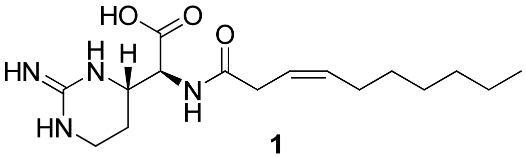

1

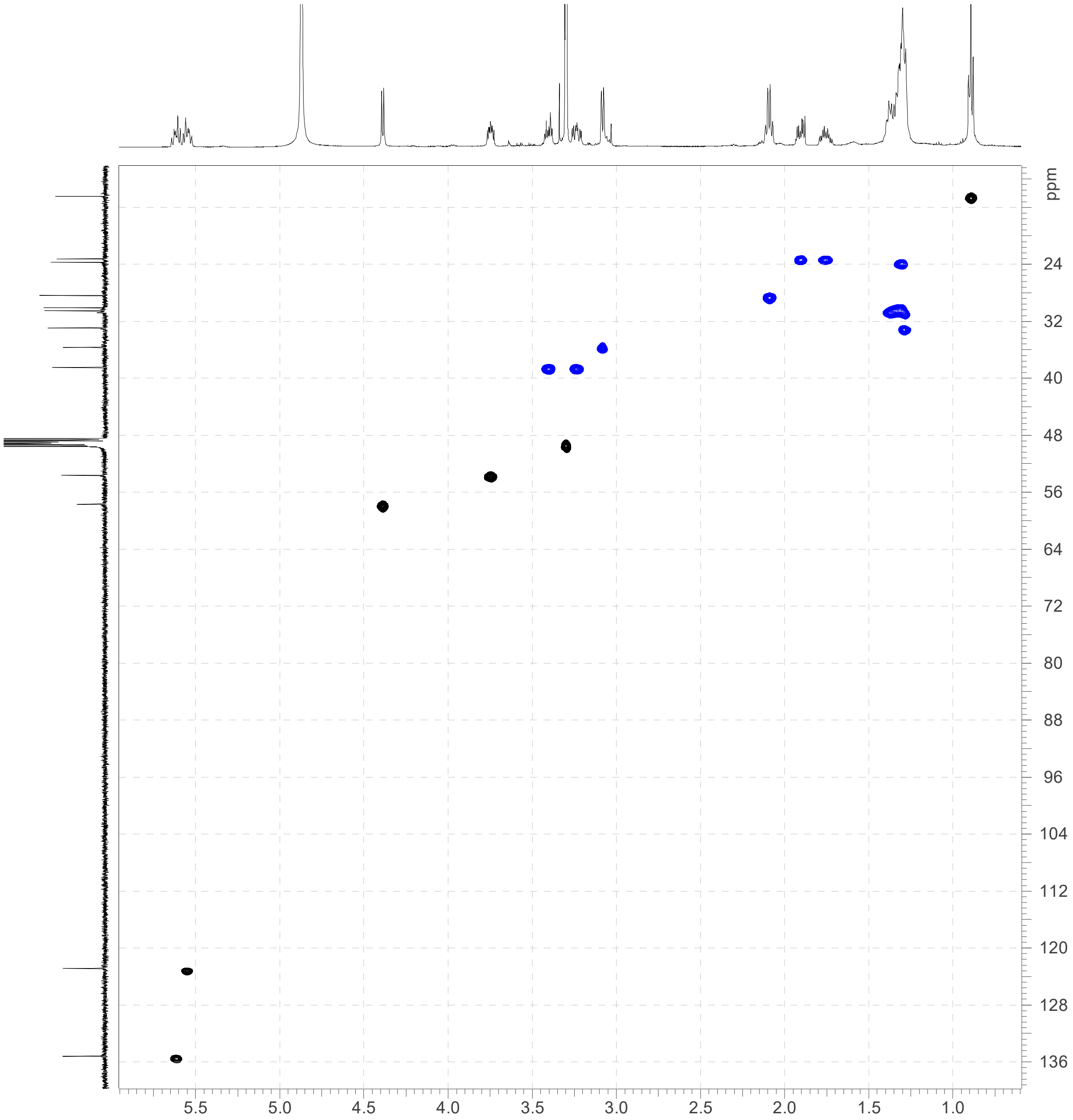

Figure S5. HSQC NMR spectrum for **1** (blue: CH_2, black: CH_3 + CH).

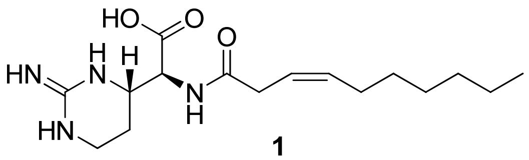

1

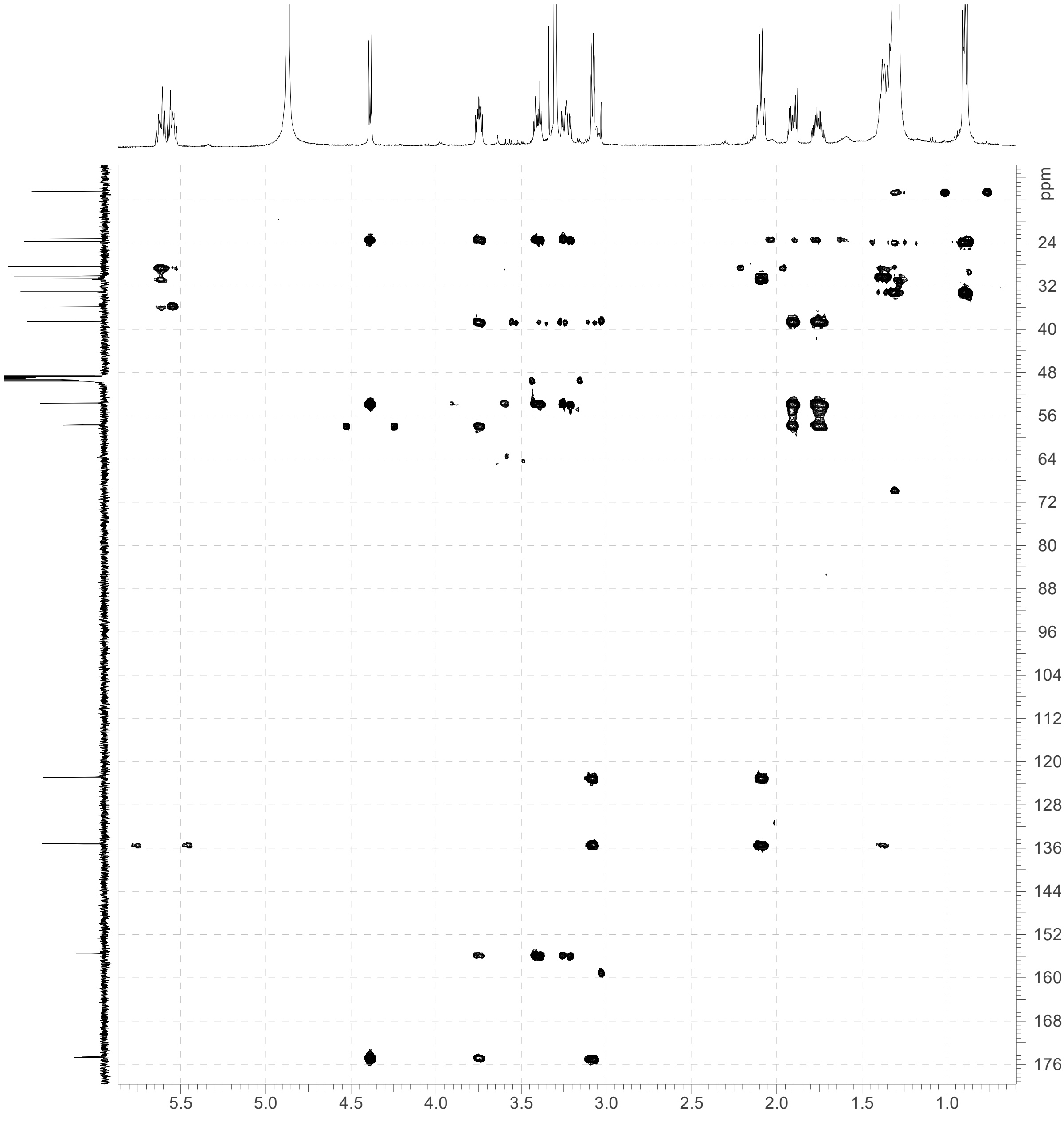

Figure S6. HMBC NMR spectrum for **1** ($^{n}J_{CH}$ = 6 Hz).

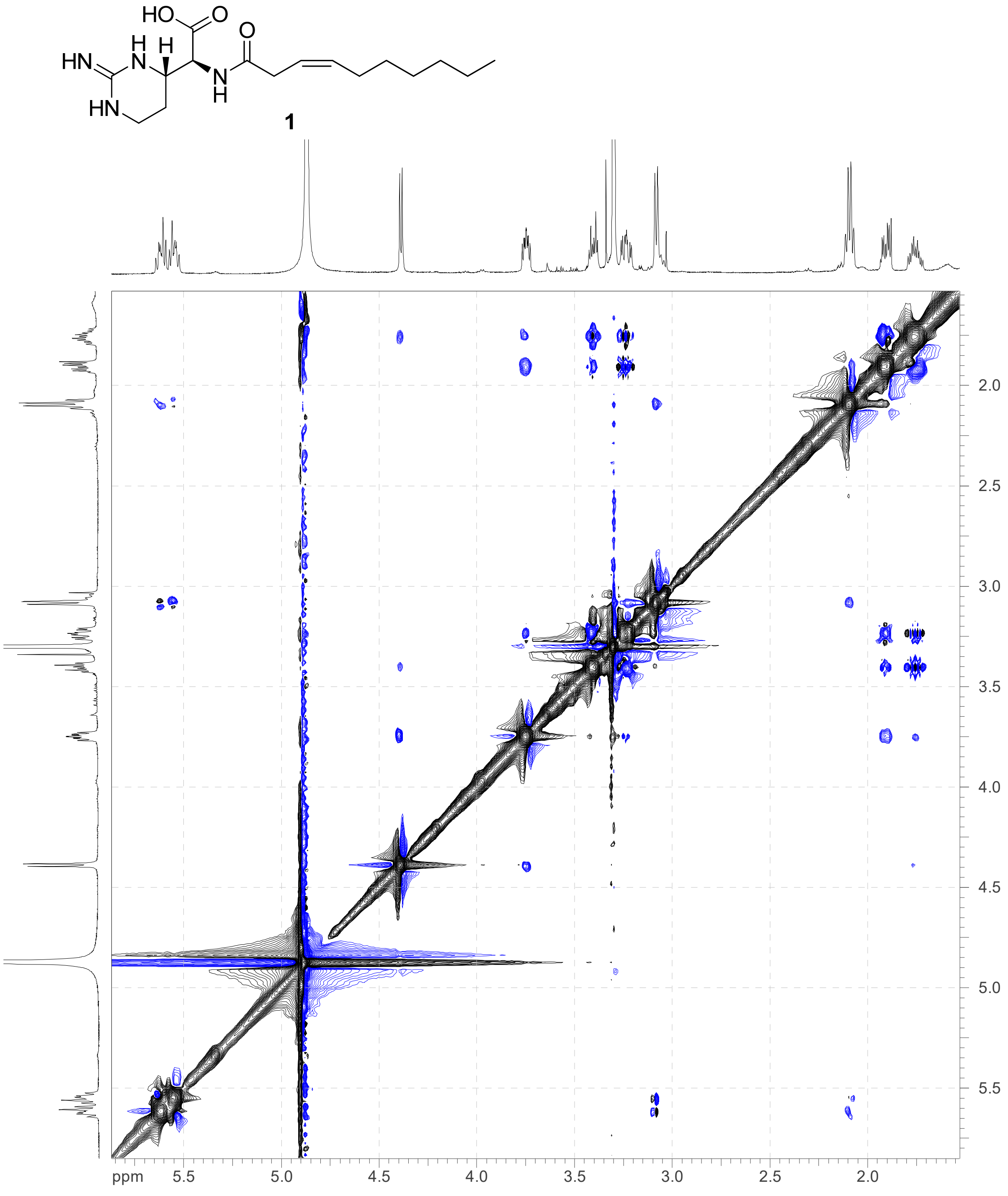

Figure S7. NOESY NMR spectrum for **1**.

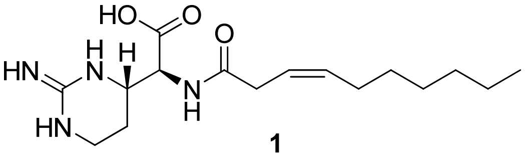

1

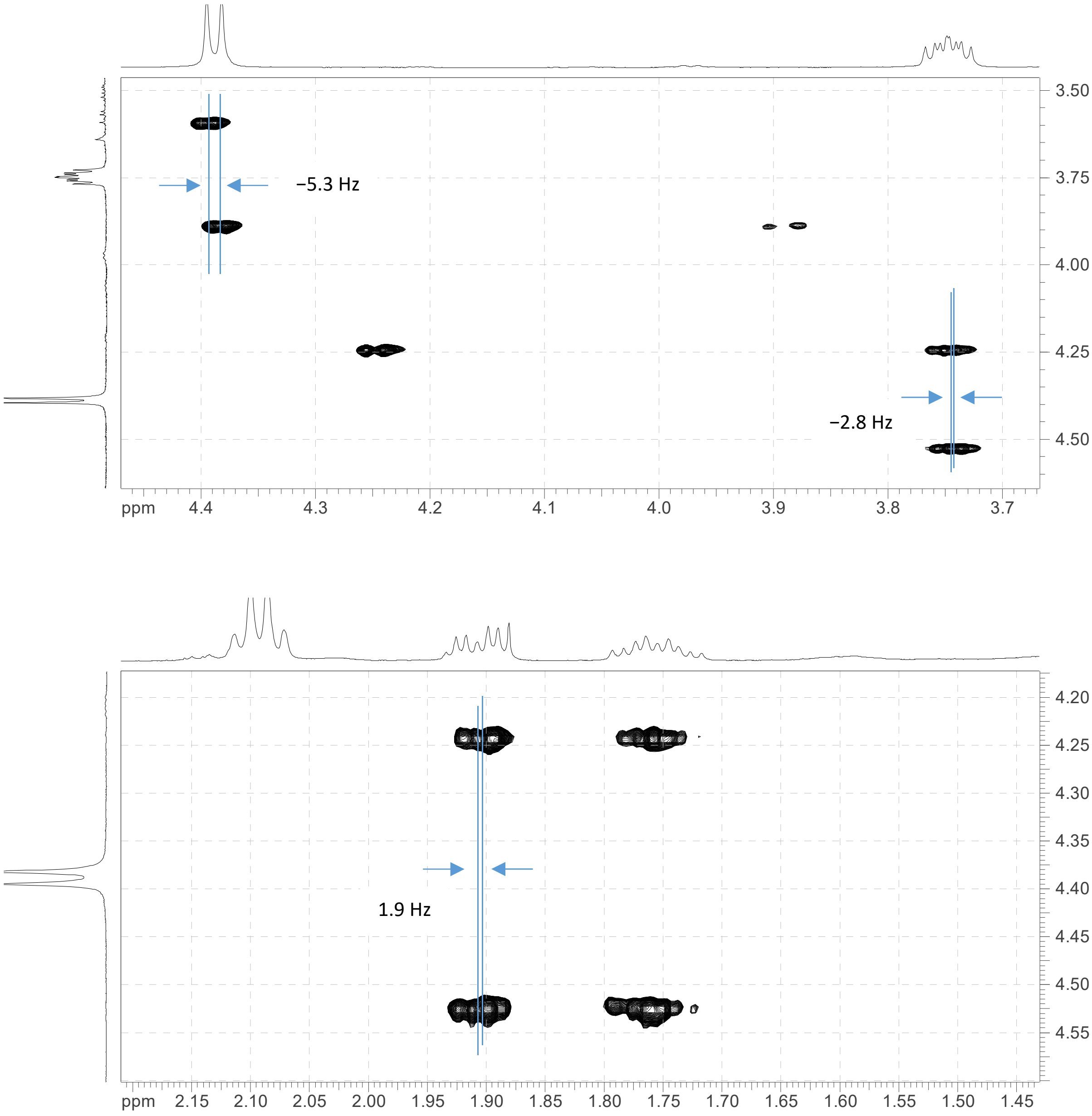

Figure S8. Expanded HETLOC NMR spectrum for **1**.

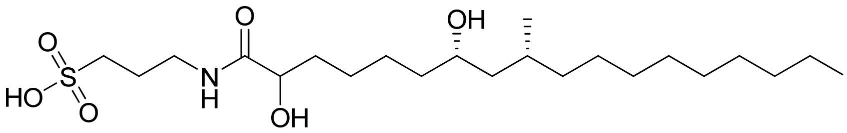

2

(A)

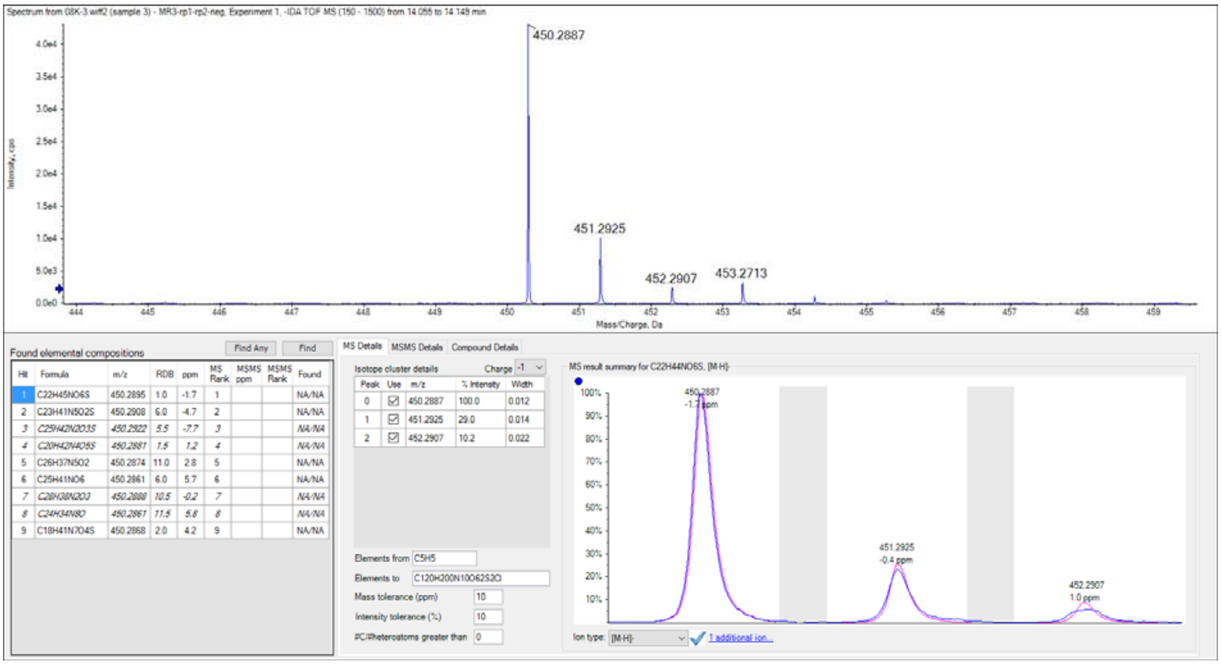

(B)

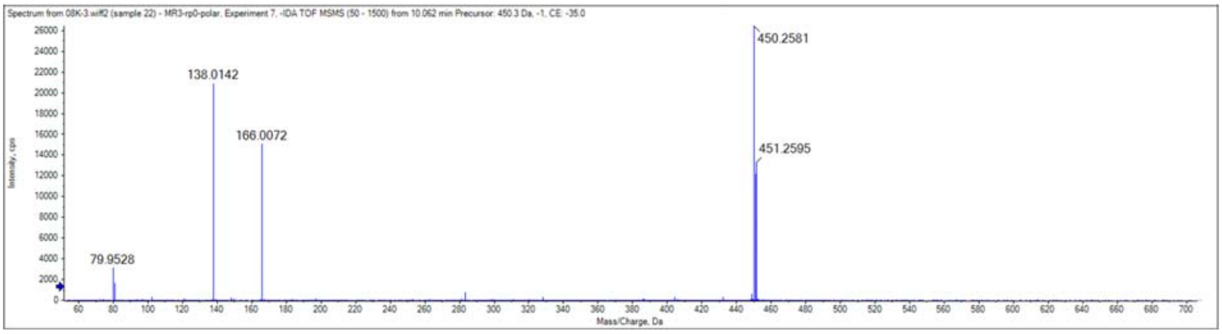

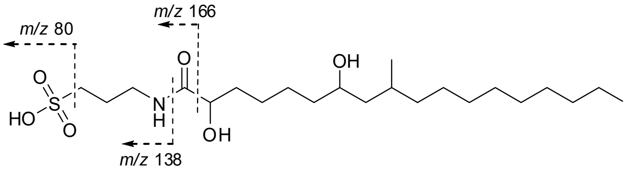

Figure S9. HRESIMS (A) and MSMS (B) data for **2**.

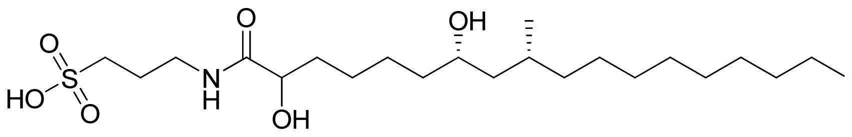

2

(A)

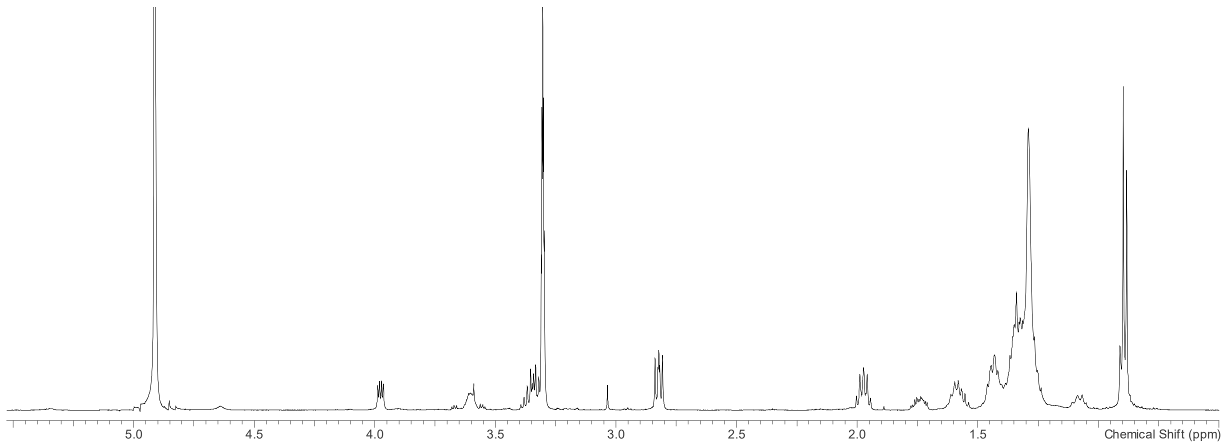

(B)

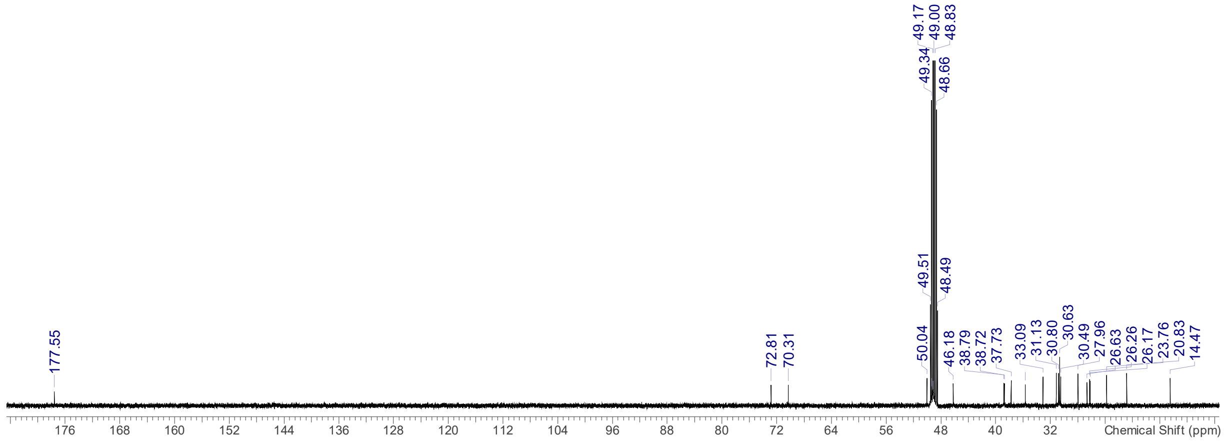

Figure S10. The ^{1}H (A) and ^{13}C (B) NMR spectra for **2**.

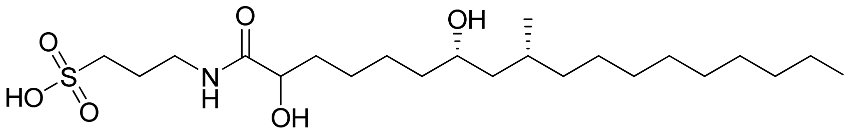

2

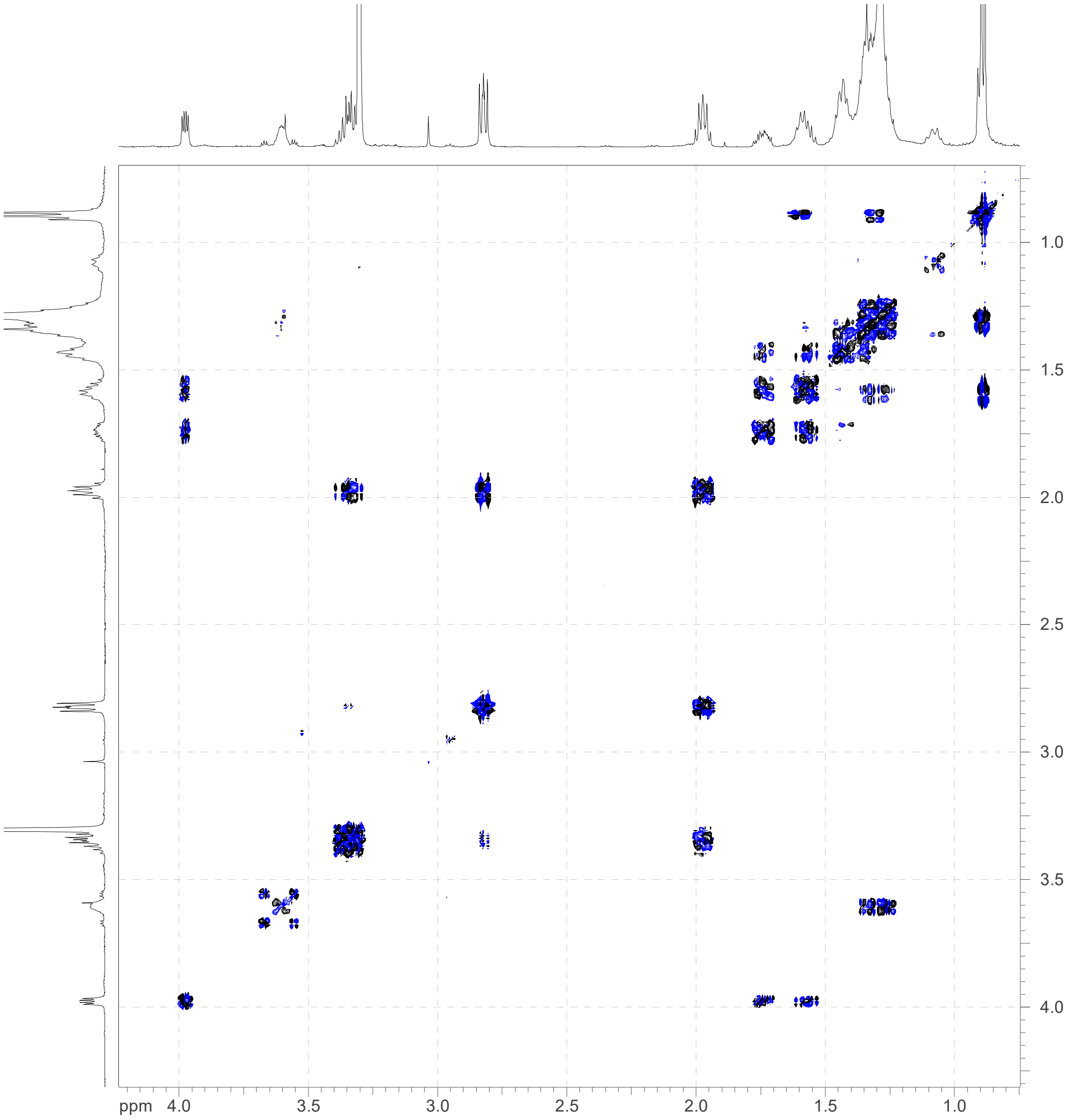

Figure S11. DQFCOSY NMR spectra for **2**.

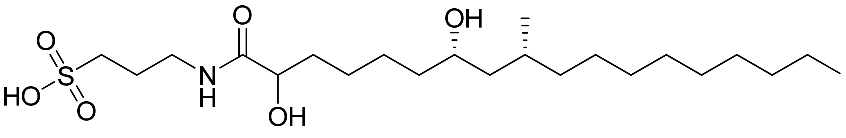

2

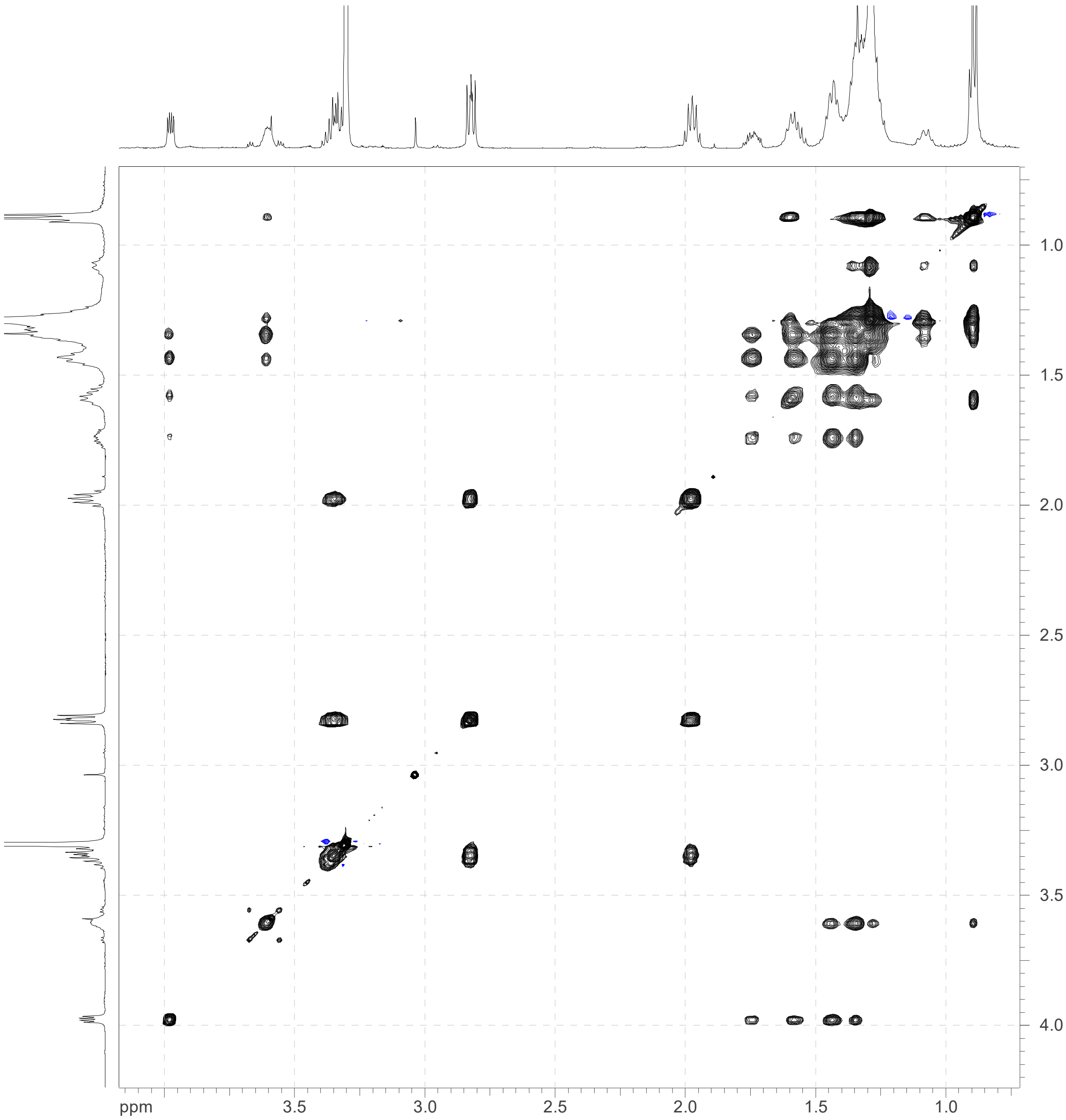

Figure S12. TOCSY NMR spectra for **2**.

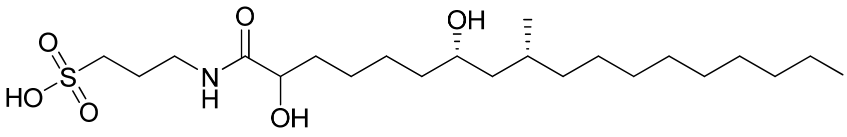

2

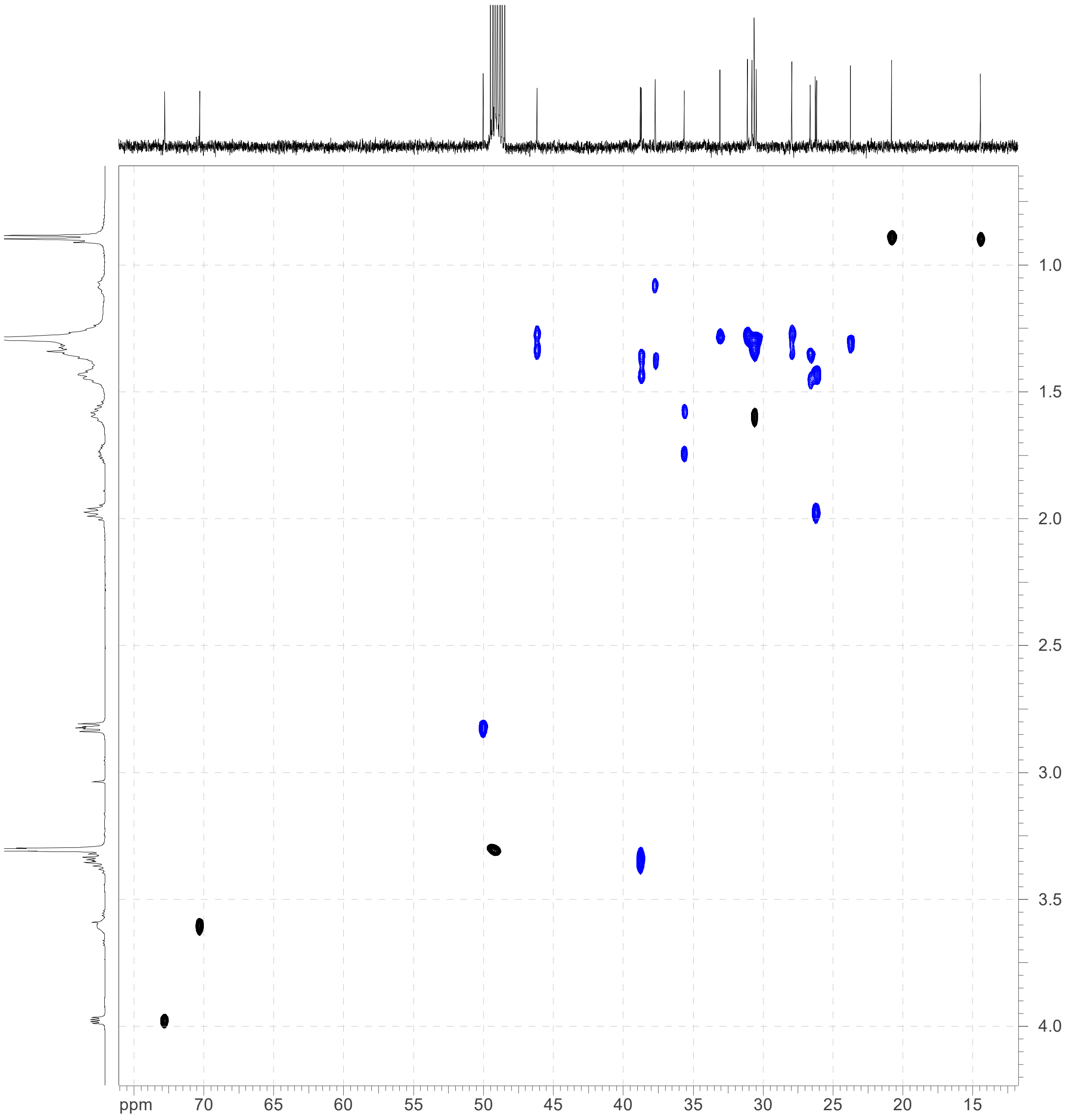

Figure S13. HSQC NMR spectrum for **2** (blue: CH_2, black: CH_3 + CH).

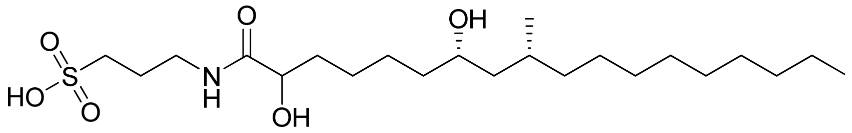

2

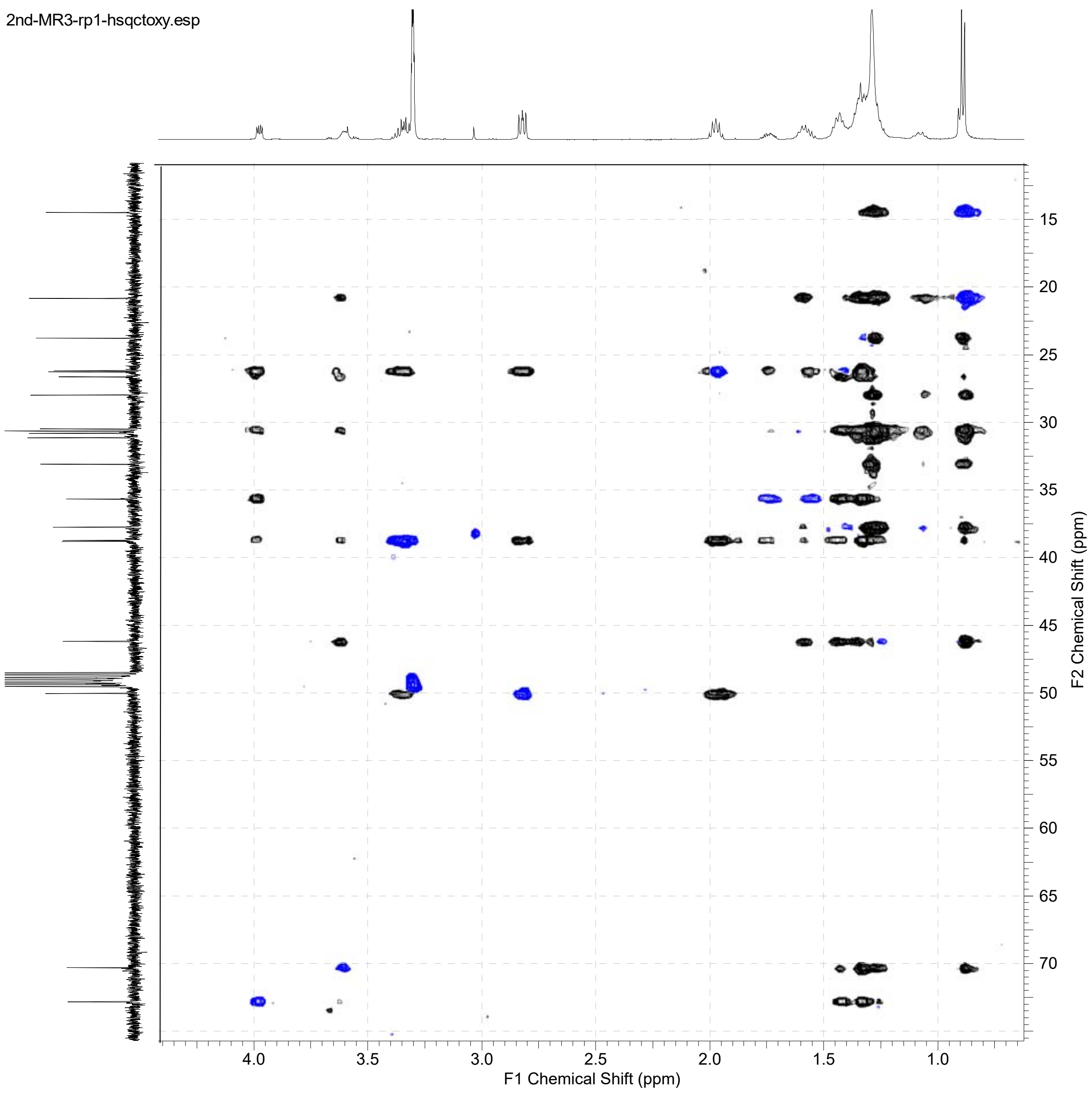

Figure S14. HSQC-TOCSY NMR spectrum for **2** (blue: $^{1}J_{CH}$, black: $^{n}J_{CH}$).

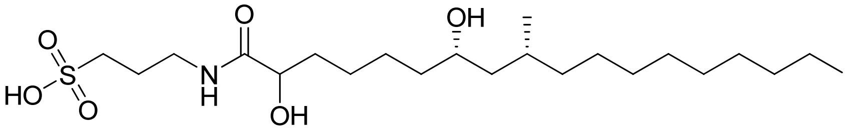

2

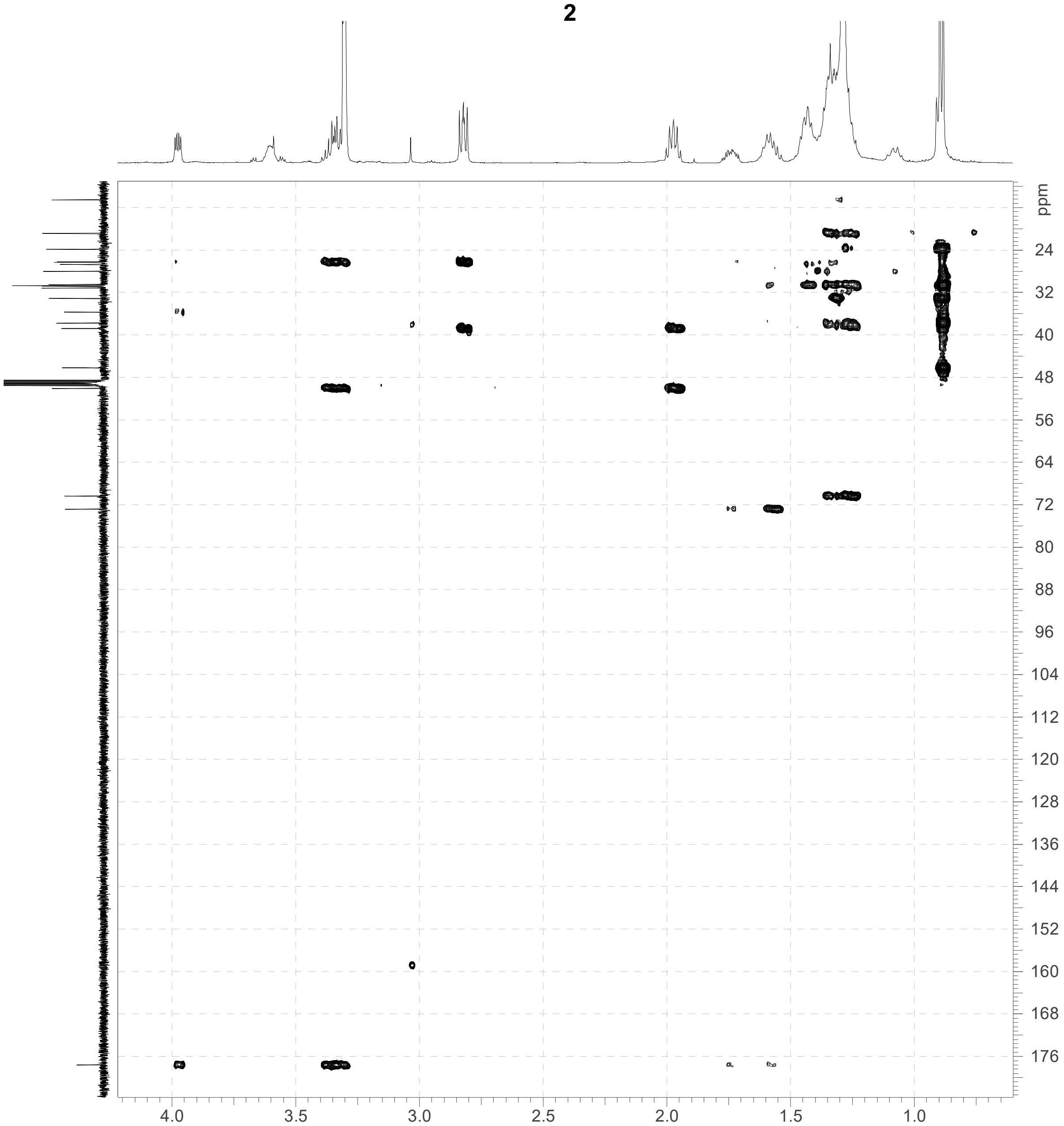

Figure S15. HMBC NMR spectrum for **2** ($^{n}J_{CH}$ = 8 Hz).

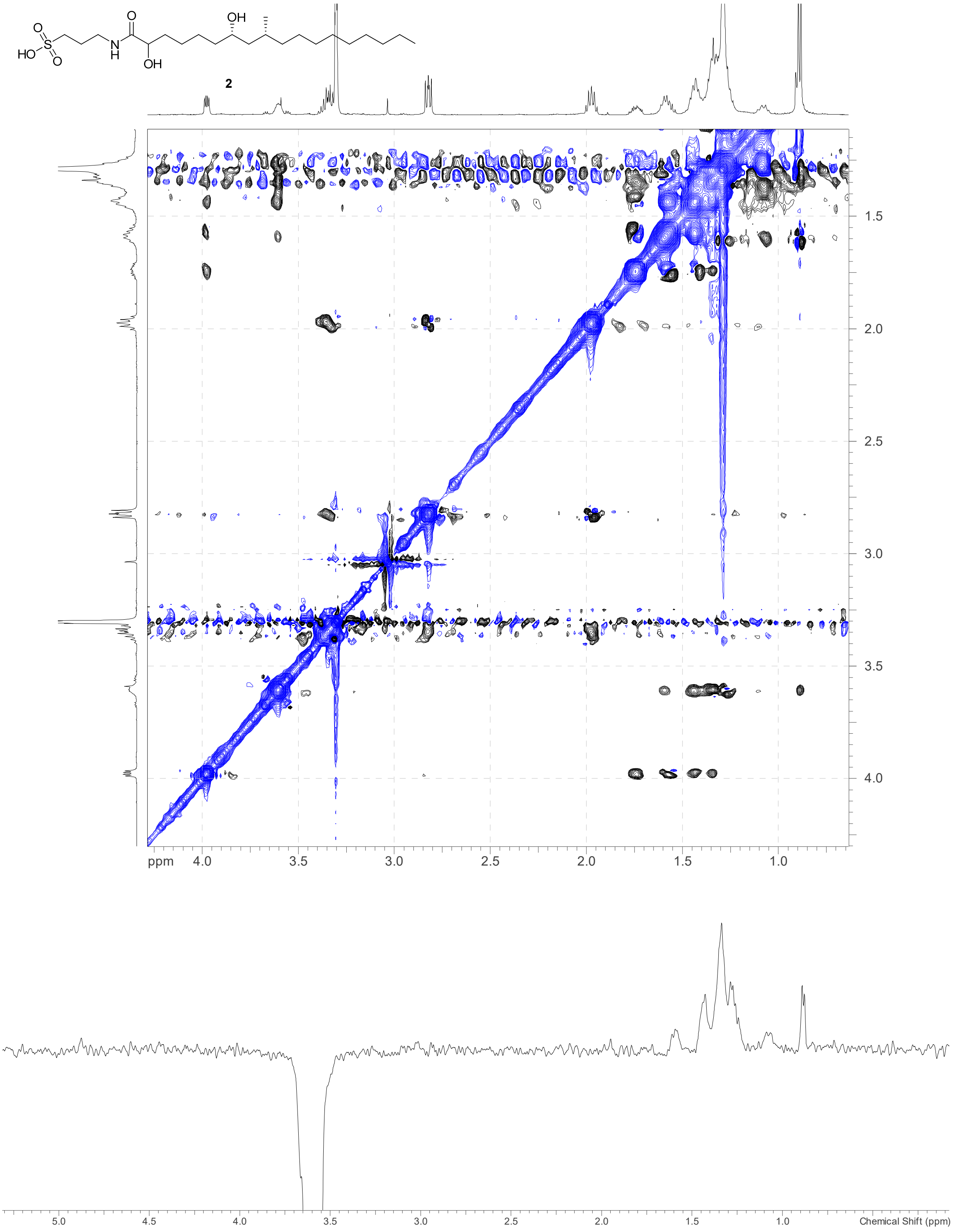

Figure S16. NOESY(up) and NOESY 1D (down) NMR spectrum for **2**.

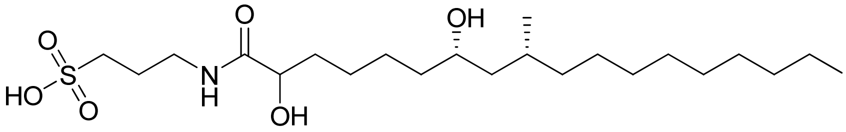

2

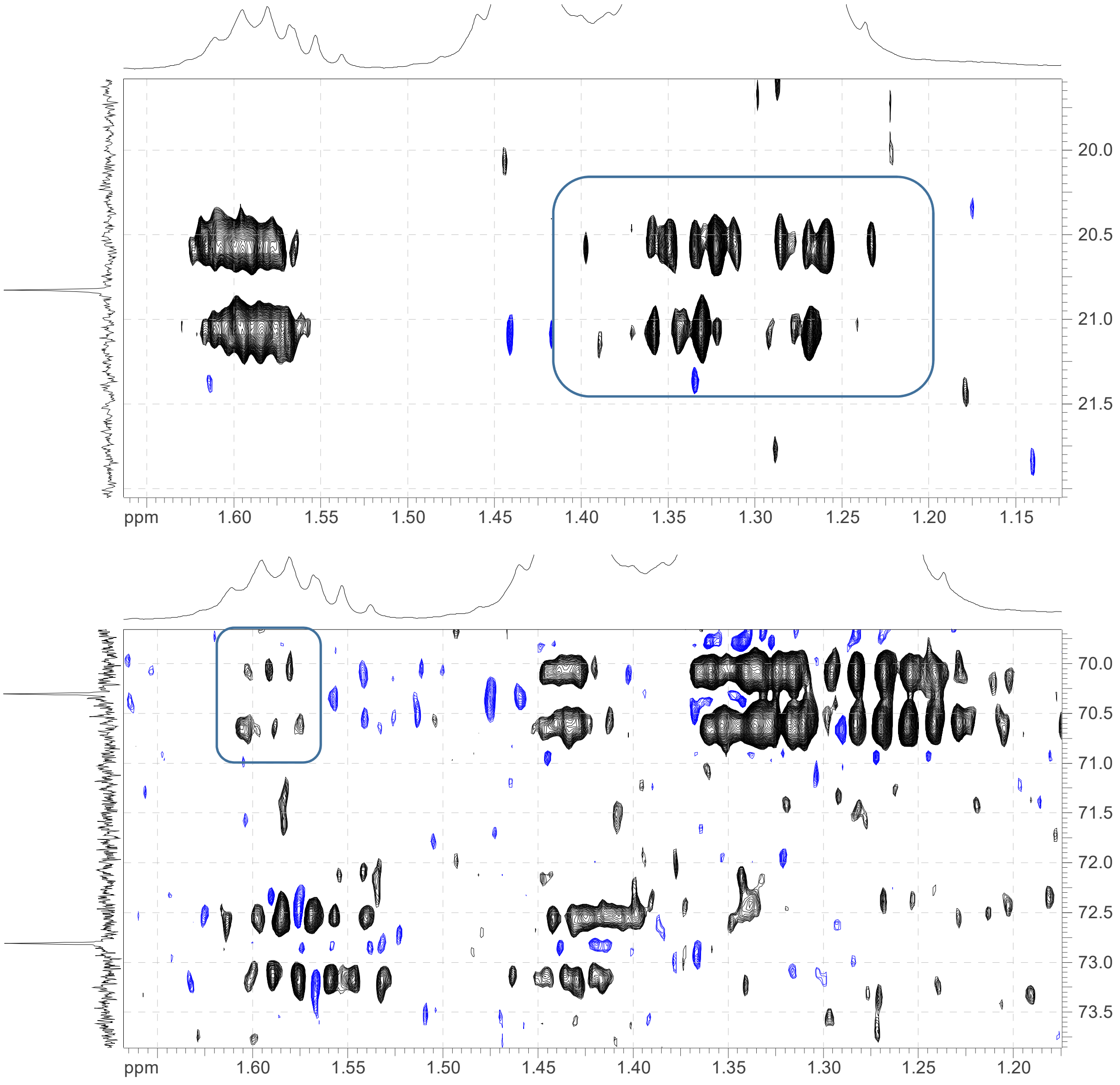

Figure S17. Expanded HECADE NMR spectrum for **2**.

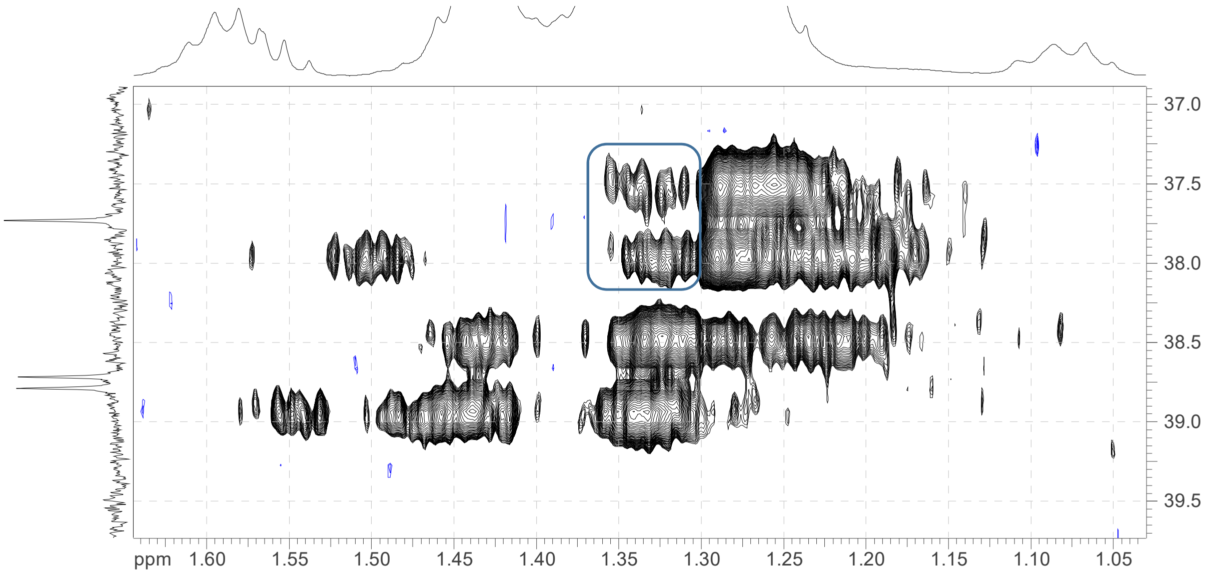

Figure S17 (Continued). Expanded HECADE NMR spectrum for **2**.
